# Preconditioning prefrontal connectivity using transcranial direct current stimulation and transcranial magnetic stimulation

**DOI:** 10.3389/fnhum.2022.929917

**Published:** 2022-08-11

**Authors:** Isabel Alkhasli, Felix M. Mottaghy, Ferdinand Binkofski, Katrin Sakreida

**Affiliations:** ^1^Section Clinical Cognitive Sciences, Department of Neurology, University Hospital, RWTH Aachen University, Aachen, Germany; ^2^Department of Nuclear Medicine, University Hospital, RWTH Aachen University, Aachen, Germany; ^3^Department of Radiology and Nuclear Medicine, Maastricht University Medical Center (MUMC+), Maastricht, Netherlands; ^4^Research Centre Jülich, Institute of Neuroscience and Medicine (INM-4), Jülich, Germany; ^5^JARA—BRAIN (Translational Brain Medicine), Jülich and Aachen, Germany; ^6^Department of Neurosurgery, University Hospital, RWTH Aachen University, Aachen, Germany; ^7^Department of Psychiatry, Psychotherapy and Psychosomatics, University Hospital, RWTH Aachen University, Aachen, Germany

**Keywords:** TMS, tDCS, DLPFC, preconditioning, resting state fMRI, functional connectivity

## Abstract

Transcranial direct current stimulation (tDCS) and transcranial magnetic stimulation (TMS) have been shown to modulate functional connectivity. Their specific effects seem to be dependent on the pre-existing neuronal state. We aimed to precondition frontal networks using tDCS and subsequently stimulate the left dorsolateral prefrontal cortex (lDLPFC) using TMS. Thirty healthy participants underwent excitatory, inhibitory, or sham tDCS for 10 min, as well as an excitatory intermittent theta-burst (iTBS) protocol (600 pulses, 190 s, 20 × 2-s trains), applied over the lDLPFC at 90% of the individual resting motor threshold. Functional connectivity was measured in three task-free resting state fMRI sessions, immediately before and after tDCS, as well as after iTBS. Testing the whole design did not yield any significant results. Analysis of the connectivity between the stimulation site and all other brain voxels, contrasting only the interaction effect between the experimental groups (excitatory vs. inhibitory) and the repeated measure (post-tDCS vs. post-TMS), revealed significantly affected voxels bilaterally in the anterior cingulate and paracingulate gyri, the caudate nuclei, the insula and operculum cortices, as well as the Heschl’s gyrus. *Post-hoc* ROI-to-ROI analyses between the significant clusters and the striatum showed post-tDCS, temporo-parietal-to-striatal and temporo-parietal-to-fronto-cingulate differences between the anodal and cathodal tDCSgroup, as well as post-TMS, striatal-to-temporo-parietal differences between the anodal and cathodal groups and frontostriatal and interhemispheric temporo-parietal cathodal-sham group differences. Excitatory iTBS to a tDCS-inhibited lDLPFC thus yielded more robust functional connectivity to various areas as compared to excitatory iTBS to a tDCS-enhanced DLPFC. Even considering reduced statistical power due to low subject numbers, results demonstrate complex, whole-brain stimulation effects. They are possibly facilitated by cortical homeostatic control mechanisms and show the feasibility of using tDCS to modulate subsequent TMS effects. This proof-of-principle study might stimulate further research into the principle of preconditioning that might be useful in the development of protocols using DLPFC as a stimulation site for the treatment of depression.

## Introduction

The notion of homeostatic plasticity in the neuronal system has been studied in animal research using electrical microstimulation for about 30 years (Iriki et al., 1989; Bear and Malenka, 1994; Hess and Donoghue, 1996). The neuronal system strives to keep cortical excitability at a physiologically optimal level by adjusting to internal and external inputs in the form of short- as well as long-term potentiation (STP/LTP) or depression (STD/LTD). Whereas STP/STD works through the temporal change of presynaptic processes that causes a change in the firing threshold of the neuron and only lasts for about 15 min, LTP/LTD is the persistent increase in synaptic strength between neurons. This mechanism of plasticity of the brain also affects communication between larger cell clusters and thus plays an important role in motor learning and memory (Asanuma and Keller, 1991; Rioult-Pedotti et al., 2000).

There have been numerous studies indicating the possibility of manipulating homeostatic plasticity using non-invasive brain stimulation methods. Siebner, Lang, and colleagues (Lang et al., 2004; Siebner et al., 2004) reported the first demonstration of the mechanism of homeostatic plasticity in the human primary motor cortex. They showed that the effects of transcranial magnetic stimulation (TMS) can be amplified by a preconditioning transcranial direct current stimulation (tDCS). Interestingly they documented a paradoxical effect: both high-frequency TMS, which is known to have a facilitating effect, but also a low-frequency TMS protocol, which is typically inhibitory, were able to amplify cortical excitability when applied to the motor hand area that was preconditioned with cathodal (inhibitory) tDCS or hamper cortical excitability when applied after anodal (excitatory) tDCS. The effect of the inhibitory or excitatory tDCS on corticospinal excitability, measured as the amplitude of motor-evoked potentials (MEPs) induced by a single TMS pulse applied to the cortical hand area and derived from one hand muscle, was thus inversed in polarity by any of the tested TMS protocols. This phenomenon has since been replicated repeatedly in the motor system, and its physiological mechanisms are well understood (Fregni and Pascual-Leone, 2007; Fricke et al., 2011; Cosentino et al., 2012). For example, Grüner et al. (2010) were able to decrease involuntary movements of the fingers and hands in patients with Parkinson’s disease using inhibitory tDCS and TMS of the primary motor cortex. Carvalho et al. (2015) were able to enhance working memory performance using tDCS preconditioning with different polarities and thus demonstrated that the polarity effect of tDCS is dependent on the precondition of the neuronal population and that stimulation effects are functionally significant in the memory domain. However, some previous neuroanatomical and electrophysiological studies have aimed at the mechanism of the modulation of the prefrontal function and found it to be much more complex when compared to the primary motor cortex (Nahas et al., 2001; Kähkönen et al., 2005; Keeser et al., 2011; Tremblay et al., 2014; Alkhasli et al., 2019). Transferring the concept of preconditioning to the prefrontal cortex would open the possibility of systematically enhancing desired plasticity changes in terms of cortical activity and through functional connectivity of interconnected networks and subcortical regions.

Repetitive TMS (rTMS) has been shown to be able to increase as well as decrease synaptic excitability in a focal cortical area (Sparing and Mottaghy, 2008). Even though rTMS does not directly influence subcortical areas, numerous studies showed rTMS induced changes in cortical-subcortical functional connectivity (e.g., Siebner et al., 1998; Paus et al., 2001; Bestmann et al., 2004; Fox et al., 2012; Shafi et al., 2012) tDCS, on the other hand, works through the placement of two electrodes on the scalp inducing a non-focal current in the brain. Rather than triggering an acute action potential in the neurons, as seen as a result of TMS, tDCS causes subtle negative (anodal) or positive (cathodal) shifts in the membrane polarity. The notion of functional connectivity is a simple measure of synchrony between BOLD-time courses of single voxels or regions of interest. It is commonly expressed as a Fisher-z transformed correlation value and has been used as a measure of communication between neuronal clusters, as it is argued that high synchrony of neuronal firing indicates a functional connection even in the absence of direct anatomical links.

The dorsolateral prefrontal cortex (DLPFC) has been shown to play a central role in the cognitive control of behaviour and other executive functions, such as attention, motor planning, procedural memory, as well as reward and emotion. Its various cortical and subcortical interconnections make it an ideal target for non-invasive brain stimulation studies (Guse et al., 2010; Dedoncker et al., 2016). It is functionally interconnected with the orbitofrontal cortex, large parts of the neocortex, the parietal cortex, the cingulate cortex, and the subcortical basal ganglia, thalamus, and hippocampus (Alexander et al., 1986; Petrides and Pandya, 1999; Tekin and Cummings, 2002; Tik et al., 2017). Animal studies demonstrated that the frontal cortex controls the release of dopamine in the striatum (Murase et al., 1993; Karreman and Moghaddam, 1996; Keck et al., 2002; Kanno et al., 2004). Disruption in the balance of the neurotransmitter glutamate and dopamine in the striatum has been observed to cause aggressive and impulsive behaviour and has been linked to neurodegenerative diseases, such as Parkinson’s disease and dementia syndrome, as well as to psychiatric diseases such as schizophrenia (Carlsson and Carlsson, 1990; Strafella et al., 2005). In a landmark study using TMS and positron emission tomography (PET), Strafella et al. showed that in human subjects, dopamine release was increased following the stimulation of the DLPFC (Strafella et al., 2001) as well as the left primary motor cortex (Strafella et al., 2003). This connection opens the possibility for the potential therapeutic use of preconditioning and modulating the DLPFC *via* non-invasive brain stimulation. Other areas affected by excitatory rTMS stimulation of the lDLPFC are mainly the anterior cingulate cortex, the amygdala in the functional connectivity domain (Paus et al., 2001; Tik et al., 2017; Alkhasli et al., 2019) but also the orbitofrontal cortex (Cho and Strafella, 2009), the insula, and the parahippocampal cortex (Sibon et al., 2007) in metabolic imaging research, and the ventromedial prefrontal cortex in a principal component fMRI analysis (Li et al., 2004).

This study is aimed at answering the question of whether the concept of preconditioning can be transferred to the human prefrontal cortex and whether an effect can be observed in terms of functional connectivity patterns between the stimulation site and its connected areas. Our goal was to utilise homeostatic plasticity in the prefrontal cortex to explore whole-brain connectivity and, more specifically, fronto-cortical and frontostriatal connectivity in task-free resting state functional magnetic resonance imaging (rsfMRI). Functional connectivity can be measured using Pearson correlation of the BOLD timelines of two regions of interest (ROIs) and expresses to what extent their communication is coupled. Here, we aimed to precondition the excitatory iTBS stimulation of lDLPFC by using either excitatory, inhibitory, or sham tDCS. At baseline, after the tDCS, as well as after a subsequent excitatory 190s long intermittent theta-burst stimulation (iTBS) protocol, we compared frontostriatal functional connectivity between the three differently preconditioned groups of participants using rsfMRI. We aimed at the question of whether homeostatic plasticity can be used to precondition and enhance or hinder functional connectivity of the frontostriatal network using tDCS and TMS. In this respect, we analysed the differential effect of anodal, cathodal, and sham tDCS on the functional connectivity of the brain. The striatum was chosen as a ROI because of its significant functional connection with the DLPFC and its clinical significance as part of the dopaminergic nigrostriatal pathway.

## Material and Methods

### Participants

Thirty neurologically and mentally healthy, right-handed (validated by the Edinburgh Handedness Inventory (Oldfleld, 1971) participants were recruited (mean age = 25.5, SD = 5.14; 15 male). Participants were pre-screened for TMS, tDCS, and magnetic resonance imaging (MRI) exclusion criteria.

### Experimental procedure

#### Overview

**Figure 1:**
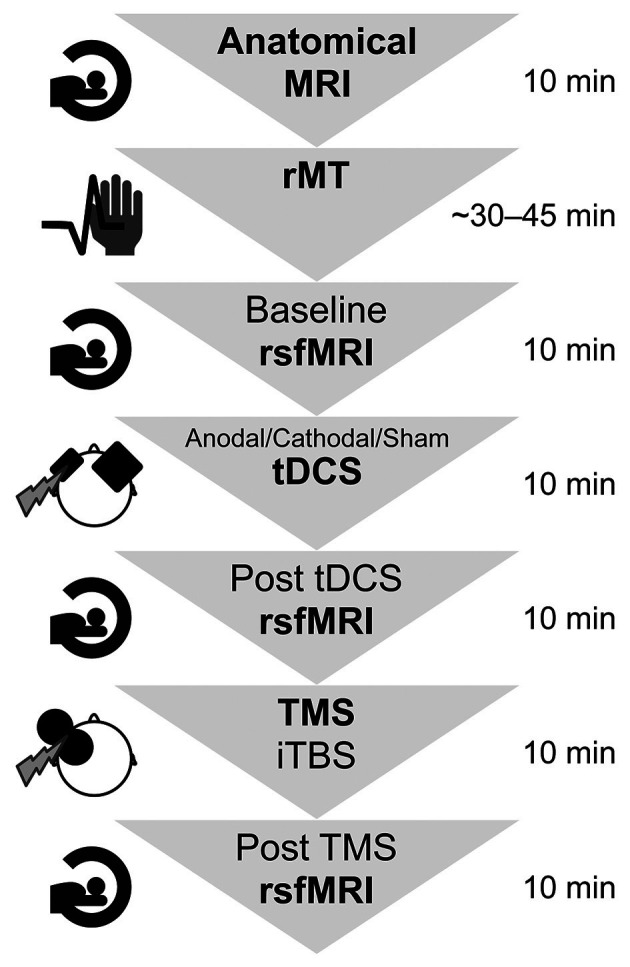
Summary of the experimental design and durations. First, the participants entered the MRI scanner for 10 min to create anatomical images. Afterwards, their individual resting motor threshold was determined, which took approximately 45 min. The experiment comprised two brain stimulation sessions as well as three functional resting state functional MRI scans lasting approximately 10 min each. The whole experimental procedure took in total approximately 2 h per participant. rsfMRI, resting state functional magnetic resonance imaging; rMT, resting motor threshold; tDCS, transcranial direct current stimulation; TMS, transcranial magnetic stimulation; iTBS, intermittent theta-burst stimulation.

An overview of the experimental procedure and the durations is shown in Figure 1. In all participants, a high-resolution anatomical MRI scan (see “TMS and MRI” Sections below) was measured, and the individual resting motor threshold (rMT) was determined using a standardised protocol (Rossi et al., 2009; Rossini et al., 2015). Afterwards, the first of three identical rsfMRI measurements were collected for each participant, lasting about 10 min. During the scan, participants were shown a small black fixation spot in the middle of a grey background. They were instructed to fixate on the dot at all times, to relax, not fall asleep, lie as still as possible, and try not to think of anything in particular. For the following application of brain stimulation by the use of tDCS and TMS, the participant was brought outside the scanner room but was lying supine on the mobile scanner bed for the entire experiment. After registration with individual anatomical MRI data (see “MRI” Section below for scanning parameters), tDCS was applied over the lDLPFC using neuronavigation (see “Transcranial direct current stimulation” Section for more details) for 10 min with either anodal (10 participants), cathodal (10 participants) polarity, or sham tDCS with either anodal or cathodal polarity (10 participants). Blinding for the tDCS protocol was assessed after all experimental procedures by asking the participants whether they knew they had received real or control stimulation and whether they had experienced any unpleasant sensations. After tDCS, participants underwent the second rsfMRI measurement before they received iTBS lasting 190s, applied over the lDLPFC using neuronavigation (see “Transcranial magnetic stimulation” Section for more details) at 90% of individual rMT. After a 7-min-break, the participants were rolled into the scanner again, lying in an unchanged position on the scanner bed, and the last 10-min-post TMS rsfMRI measurement was conducted. This 7-min interval was kept constant between all participants and was implemented to allow for arousal and discomfort ratings and MRI preparations. Immediately after each stimulation, arousal was assessed using a 9-level self-assessment manikin (SAM) scale, with level 1 corresponding to “very calm and relaxed” and level 9 corresponding to “very excited, stimulated, furious, excited, aroused” (Bradley and Lang, 1994). After TMS, an additional 5-level Likert scale was used to measure discomfort during iTBS stimulation (1 = none, 5 = strong). To test whether arousal or discomfort was significantly different between the tDCS groups or measurement points, non-parametric tests were conducted.

#### Transcranial direct current stimulation

TDCS was administered as anodal (1 mA), cathodal (−1 mA), or sham (0 mA, either anodal or cathodal electrode placement) stimulation using an MRI-compatible stimulator (neuroConn GmbH, Ilmenau, Germany). Sham stimulation was thus a zero-current stimulation that mimics the surface sensation of real tDCS but does not reach subcranial areas. Electrode placement was alternated between the participants in the sham condition in oder to avoid surface sensation effects specific to one side. The fade-in/out period was 10 s. The placement of the active electrode sized 5 by 7 cm was determined by transforming the individual anatomical images into the MNI system using the neuronavigation system (LOCALITE Biomedical Visualization Systems GmbH, Sankt Augustin, Germany) and marking the MNI coordinates (x, y, z) = −50, 30, 36 with the neuronavigation pointer as stimulation target. These coordinates were suggested by Rusjan et al. (2010) for an optimal location of the lDLPFC by neuronavigation as compared to conventional distance-based localisation methods. The tDCS reference electrode sized 10 by 10 cm was placed contralateral and supraorbital. The stimulation was applied for 10 min. Again, just as during rsfMRI, participants were instructed to relax, not fall asleep, lie as still as possible, and try not to think of anything in particular but they could close their eyes.

#### Transcranial magnetic stimulation

Both single-pulse TMS for determination of the individual rMT and the experimental iTBS was applied using a figure-of-eight coil (C-B60) connected to a MagPro X100 stimulator (MagVenture, Farum, Denmark) guided by neuronavigation. In preparation for rMT determination, the presumed hand area was identified visually through anatomical landmarks in the left motor cortex. Participants were placed in a comfortable chair or lying down on the MRI scanner bed outside the scanner room for registration with their individual anatomical MRI data. Three pre-gelled disposable surface electrodes were fitted to the participant’s right hand (first dorsal interosseous muscle, index finger, inner wrist) to derive MEPs which were monitored (MagVenture, Farum, Denmark, connected to the MagPro X100 stimulator). Biphasic single pulses were applied over the presumed hand area starting at 30% of stimulator output but were increased until clear MEPs and hand muscle contraction could be observed. In most participants approximately 5–10 pulses were applied to the motor area in order to identify the hand area. The intensity was then reduced stepwise to find the lowest intensity that induces supra-threshold (>50 μV) MEPs above chance, i.e., we used the standard rule that the rMT corresponds to the minimum stimulation intensity at which MEPs of at least 50 μV are elicited in at least 5 of 10 consecutive trials (50%) in the resting target muscle (Rossini et al., 2015).

The experimental excitatory iTBS (Huang et al., 2005) protocol consisted of 600 pulses spaced-out over 3 min and 20 s. It was comprised of 20 trains and 10 theta-bursts. Between each of the 2-s-long trains (50 Hz), there was an 8-s long pause. The lDLPFC stimulation site was determined in the same way as the tDCS target. Actual individual stimulation sites were recorded during the TMS procedure and used as subject-specific seed regions. Participants received stimulation at an intensity of 90% of their individual rMT. The mean rMT was 45.63% (*SD* = 6.82) of the maximum stimulator output, and the mean stimulation applied was 41.01% (*SD* = 5.68) of the maximum stimulator output. The stimulation threshold of 90% of the individual resting motor threshold was chosen based on related experiments by our group that found a strong frontostriatal modulation effect at that threshold (Alkhasli et al., 2019).

Simultaneously with each TMS pulse, stimulation markers, including the information of the exact position of the coil hotspot and its perpendicular projection onto the brain surface, were recorded by the neuro-navigation system. For each participant, we exported one of the first stimulation markers as the volume of interest into the NIfTI file format for further image analyses.

#### Magnetic resonance imaging

MRI scans were measured on a Magnetom Prisma 3.0 T whole-body scanner (Siemens Medical Solutions, Erlangen, Germany). Anatomical data was acquired using a three-dimensional magnetization-prepared, rapid acquisition gradient-echo sequence (MP-RAGE) with the following parameter: 300 repetitions, TR = 2,300 ms, TE = 2.98 ms, 9° flip angle, FOV = 256 mm, 176 sagittal slices, slice thickness = 1 mm and in-plane resolution = 1 × 1 × 1 mm.

RsfMRI data were measured with a gradient echo-planar imaging (EPI) sequence with the following parameters: TR = 2,000 ms, TE = 28 ms, 77° flip angle, FOV = 192 mm, 34 axial slices (interleaved acquisition), 3 mm slice thickness, echo planar imaging volumes and in-plane resolution = 3 × 3 × 3 mm. Both sequences lasted about 10 min.

MRI data were analysed using the Statistical Parametric Mapping software SPM12 (Welcome Department of Cognitive Neurosciences, London, UK) and CONN Functional Connectivity version (18.b, Whitfield-Gabrieli and Nieto-Castanon, 2012) toolboxes running under Matlab R2012b (MathWorks Inc., Natick, MA, USA). Pre-processing of the rsfMRI data included: the removal of the first five volumes to discard saturation effects, slice time correction, realignment, segmentation, nuisance covariates regression with white matter, and cerebrospinal fluid as regressors, head motion correction, head motion scrubbing as a regressor (scans with a framewise displacement >0.9 mm or global BOLD signal changes >5 SD are removed), bandpass filtering of the frequencies 0.01–0.08 Hz and linear detrending. The root-mean-square of the head motion translation parameters [displacement = square root (x^2^ + y^2^ + z^2^)] across all participants and sessions was 0.13 mm (*Max* = 0.25 mm, *SD* = 0.05 mm), with a mean subject-wise maximum of 0.97 mm (*Max* = 3.07 mm, *SD* = 0.83 mm).

#### Functional connectivity

Seed-to-voxel correlations were calculated to explore the whole brain stimulation effect. For each subject, the activity of the individual stimulation site (lDLPFC, a sphere with a diameter of 1 cm) was extracted as an unweighted mean BOLD signal change time series. Three-dimensional stimulation seed masks were obtained from each participant’s individual T1-anatomy and then co-registered with the corresponding functional data set. Functional connectivity was then calculated as a Fisher-Z-transformed correlation coefficient between the stimulation site (lDLPFC, seed) signal and all individual voxel signals separately. All correlation values are Fisher-Z-transformed. Alpha value inflation caused by multiple comparisons was corrected on a cluster-size level.

To explore connectivity patterns specifically between the stimulation site and specific ROIs, ROI-to-ROI-analyses were calculated. Functional connectivity was additionally calculated using three clusters extracted from the seed-to-voxel analysis interaction effect, as well as a bilateral striatum mask (Harvard-Oxford atlas, consisting of caudate, putamen, and nucleus accumbens). The ROIs constructed from the clusters consisted of the entire cluster (all significant voxels). Striatal signal timeseries were thus extracted from MNI normalised functional data. In total, there were four different seed masks: lDLPFC (stimulation site), the three extracted clusters, and the striatum.

Calculating a 3 × 3 mixed effect analysis of variance (ANOVA) of the functional connectivity between the stimulation site seed and each of the additional four ROIs (three clusters and striatum) resulted in four separate ANOVAs. For each ANOVA, the within-subjects effect was the repeated measure (baseline vs. post-tDCS vs. post-TMS) and the between-subjects effect was the tDCS group (anodal vs. cathodal vs. sham tDCS).

## Results

### Whole-brain seed-to-voxel analysis

To get an overview of lDLPF-to whole-brain connectivity at baseline, we first calculated a one-sample t-test for all participants at baseline. The results were FDR corrected at the voxel level and cluster size level (*α* = 0.01% FDR corrected *p*). There were 16 significant clusters. Details can be found in Table 1.

**Table 1:** lDLPFC to whole-brain results at baseline.

	**Cluster peak**		**Voxels**	**Size *p***	**Size *p***	
	**(x, y, z)**		**s**	**(FDR)**	**(unc.)**	**Anatomical Description of Peak Voxels (AAL)**
−36	40	20	1,853	>0.001	>0.001	Left Middle Frontal Gyrus
−40	26	40	1,556	>0.001	>0.001	Right Middle Frontal Gyrus
−48	−46	56	669	>0.001	>0.001	Left Inferior Parietal Gyrus
−0	22	42	486	>0.001	>0.001	Left Superior Medial Frontal Gyrus
−56	−40	54	227	>0.001	0.015	Right Inferior Parietal Gyrus
−26	2	60	139	>0.001	0.135	Left Middle Frontal Gyrus
−60	−6	−20	136	>0.001	>0.001	Left Middle Temporal Gyrus
−30	16	6	132	>0.001	0.015	Left Insula
30	4	66	113	>0.001	0.008	Right Superior Frontal Gyrus
−50	4	22	99	>0.001	0.006	Left Precentral Gyrus
−2	−48	30	84	>0.001	0.089	Left Posterior Cingulum
−36	−54	−32	71	>0.001	0.038	Left Cerebellum Crus 1
−24	48	−14	69	>0.001	0.018	Left Middle Orbital Gyrus
−6	−68	54	54	>0.001	0.119	Left Precuneus
−54	12	12	43	>0.001	0.012	Right Inferior Operculum Frontal Gyrus
−32	20	6	27	>0.001	0.254	Right Insula

*Results of baseline whole-brain seed-to-voxel analysis. To get an overview of lDLPFC, whole-brain connectivity at baseline across all participants was calculated. All clusters were then used as ROIs in a subsequent ROI-to-ROI analysis to test the whole experimental design. There were no significant connections in this 3 × 3 interaction contrast. p, p-value; unc., uncorrected; AAL, Automatic Anatomical Labelling*.

Next, to explore the effect the stimulation protocols had on each of the three experimental groups, three separate seed-to-voxel F-tests were conducted comparing lDLPF-to whole-brain functional connectivity between the baseline, post tDCS, and post TMS. Results were corrected using uncorrected voxel threshold and false discovery rate-corrected (FDR, Benjamini and Hochberg, 1995) cluster-size (*α* = 5%, *df* = 8). There were no significant clusters for the anodal and cathodal groups. However, there was one significant cluster of 36 voxels covering the right precuneus (peak MNI coordinates: x, y, z = 10, −62, 28) in the sham group. Mean functional connectivity of this precuneus seed was 0.01 (SD = 0.15) at baseline, −0.15 (SD = 0.12) post tDCS and −0.22 (SD = 0.18) post TMS.

**Figure 2:**
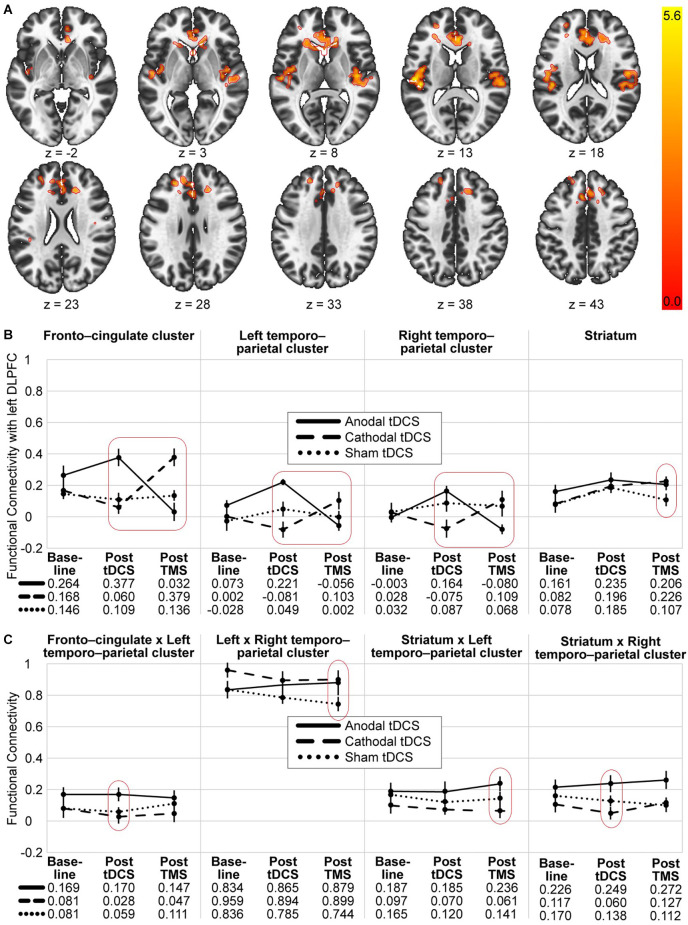
Visualisation of the seed-to-voxel and ROI-to-ROI analysis results. **(A)** Significant voxel and cluster location for the seed-to-voxel analysis with the 2 × 2 contrast: anodal > cathodal and post tDCS > post-TMS. The coloured scale indicates T-values of significant voxels. **(B)** Each diagram represents the mean functional connectivity in the three groups (anodal, cathodal and sham tDCS) and three timepoints (baseline, post tDCS and post TMS) and between the stimulation site and three significant clusters and the striatum, respectively. Only the entire design, the repeated measure differences of the sham group and planned *post-hoc* between-group-comparisons at each of the post stimulation timepoint were tested. **(C)** Diagrams of mean functional connectivity values for each group and timepoint off our additional significant ROI-to-ROI connections. Significant interactions are marked with a red square and significant differences are encircled (see Tables s 2, 3 for details). Error bars represent standard errors. Anodal tDCS, solid line; cathodal tDCS, dashed line; sham tDCS, dotted line.

In order to identify clusters significantly affected by the interaction of the two experimental stimulations (post-tDCS and post-TMS), an exploratory seed-to-voxel analysis was conducted. Contrasting only the interaction effect between the two factors “preconditioning” (anodal tDCS vs. cathodal tDCS) and “timepoint of post-stimulation measurement” (post-tDCS vs. post-TMS) did yield three significant clusters [*α* = 5% uncorrected voxel threshold and false discovery rate (FDR, Benjamini and Hochberg, 1995)-corrected cluster-size, *α* = 5% *df* = 18]. A visualisation and summary of anatomical and statistical properties of the significant clusters can be found in Table 2 and Figure 2. There was a large bilateral cluster of 1,638 voxels that covered mostly the cingulate and paracingulate gyrus, the frontal pole and small portions of the caudate in the subcortical basal ganglia. The two other smaller clusters covered large parts of the insula and operculum and the Heschl’s gyrus in both hemispheres.

**Table 2:** ROI-to-voxel analysis: interaction-contrast results.

**Cluster peak**	**Voxels**	**Size *p***	**Size *p***		**Anatomical Description**	
**(x, y, z)**		**(FDR)**	**(unc.)**	**Voxels**	**Region**	**% of Region**
**Fronto-cingulate cluster**
**4, 30, 14**	1,638	<0.001	<0.001	399	anterior cingulate gyrus	15
				144	l. frontal pole	2
				128	r. paracingulate gyrus	9
				121	l. paracingulate gyrus	9
				75	r. superior frontal gyrus	3
				23	l. caudate	4
				10	r. frontal pole	<1
				8	l. superior frontal gyrus	<1
				8	r. caudate	2
**Left temporo-parietal cluster**
**−44, −26, 14**	479	0.015	<0.001	215	l. central opercular cortex	22
				100	l. Heschl’s gyrus	32
				69	l. insular cortex	5
				66	l. parietal operculum	12
				12	l. planum temporale	2
				1	l. planum polare	<1
**Right temporo-parietal cluster**
**42, −16, 8**	583	0.006	<0.001	105	r. insular cortex	8
				103	r. planum temporale	23
				101	r. central opercular cortex	12
				90	r. parietal operculum	17
				83	r. Heschl’s Gyrus	29
				6	r. postcentral gyrus	<1
				1	r. anterior supramar. gyrus	<1

Results of ROI-to-voxel analysis. A 2 × 2 contrast (Anodal vs. cathodal and post-tDCS vs. post-TMS) was calculated between the mean signal of the spherical stimulation site seed and each individual voxel of the rest of the whole brain masks. The alpha level was kept at 5% (uncorrected on the voxel level and FDR-corrected on the cluster level for multiple comparisons). Three big clusters were significant. The number of voxels, as well as an anatomical classification, was done automated by the Matlab/SPM toolbox CONN based on the Harvard-Oxford and AAL atlas are listed. Numbers in the last column indicate the percentage of significant voxels of the atlas region listed. p, p-value; l., left; r., right.

**Table 3:** Results of planned *post-hoc* T-tests of ROI to ROI data.

Timepoints	Groups	Statistics	p-FDR	Sig. connections
**Baseline ×**	**Sham × Anodal ×**	*F*_(4,52)_	<0.0500	none
**post tDCS ×**	**Cathodal**		
**post TMS**	
**Baseline ×**	**Sham**	*F*_(2,8)_	<0.0500	none
**post tDCS ×**			
**post TMS**			
**Post tDCS**	**Anodal >**	*F*_(2,17)_ = 10.13	0.0038	**Cluster 1**
	**Cathodal**	*T*_(18)_ = 5.21	0.0002	lDLPFC × l. temporo-parietal cluster
		*T*_(18)_ = 3.45	0.0038	lDLPFC × r. temporo-parietal cluster
		*T*_(18)_ = 2.66	0.0632	Striatumx r. temporo-parietal cluster
		*T*_(18)_ = 2.16	0.0887	Fronto-cingulate × l. temporo-parietal cluster
		*F*_(2,17)_ = 6.67	0.0109	**Cluster 2**
		*T*_(18)_ = 4.35	0.0008	lDLPFC × Fronto-cingulate cluster
	**Anodal > Sham**	*F*_(2,17)_ = 5.80	0.0361	**Cluster 1**
		*T*_(18)_ = 3.62	0.0079	lDLPFC × Fronto-cingulate cluster
	**Cathodal > Sham**	*T*(18)	<0.0500	None
**Post TMS**	**Anodal >**	*F*_(2,17)_ = 7.83	0.0117	**Cluster 1**
	**Cathodal**	*T*_(18)_ = −4.07	0.0029	lDLPFC × Fronto-cingulate cluster
		*F*_(2,17)_ = 5.96	0.0164	**Cluster 2**
		*T*_(18)_ = −2.77	0.0255	lDLPFC x r. temporo-parietal cluster
		*T*_(18)_ = −2.32	0.0433	lDLPFC x l. temporo-parietal cluster
		*T*_(18)_ = 2.52	0.0863	Striatum x l. temporo-parietal cluster
	**Anodal > Sham**	*T*(18)	<0.0500	none
	**Cathodal > Sham**	*F*_(2,17)_ = 7.40	0.0146	**Cluster 1**
		*T*_(18)_ = 3.54	0.0094	lDLPFC x Fronto-cingulate cluster
		*T*_(18)_ = 2.25	0.1484	lDLPFC x striatum
		*F*_(1,18)_ = 5.78	0.0407	**Cluster 2**
		*T*_(18)_ = 2.40	0.1087	L. x r. temporo-parietal cluster

*Results of ROI-to-ROI analysis. ANOVAs and *post-hoc* t-tests were calculated to test functional network connectivity between the mean signal of the five ROIs. The alpha level was kept at 5% (FDR-corrected on the cluster level and uncorrected on the connection level). Additional connections that were not revealed by the seed-to-voxel analysis are marked by an underscore. Those were a striatal-right temporo-parietal and fronto-cingulate-left temporo-parietal difference between the anodal and cathodal groups in the post tDCS and striatal-left temporo-parietal differences between the anodal and cathodal groups in the post-TMS measurements as well as a left-right temporo-parietal difference between the cathodal and sham group post-TMS. p., p-value; L./l. left; r., right*.

### Region of interest analysis

To answer the question whether connectivity between areas that were functionally connected to the stimulation site at baseline differed in their reaction to the experimental stimulations between the groups and timepoints, we extracted the 16 baseline clusters of the seed-to-voxel-analysis and calculated a 3 × 3 MANOVA on ROI-to-ROI functional connectivity between these 16 clusters and an lDLPFC and a striatum seed. There were no significant clusters at an *α* = 5% (connection threshold uncorrected and cluster level FDR corrected, multivoxel pattern analysis).

To visualise and further analyse the interaction effects of the experimental groups, especially the involvement of the striatum, ROI-to-ROI analysis was conducted using the following five ROIs: lDLPFC (stimulation site), fronto-cingulate cluster (x, y, z = 4, 30, 14), left temporo-parietal cluster (x, y, z = −44, −26, 14, right temporo-parietal cluster (x, y, z = 42, −16, 8) and striatum. Mean ROI-to-ROI functional connectivity was extracted using CONN and visualised in Figure 2A. A summary of all ROI-to-ROI analysis results can be found in Table 3. First, to test whether there was a significant effect of the whole experimental design on the functional connectivity between the five ROIs, an FDR-corrected, 3 × 3 ANOVAs for all ROI connections was conducted [*n* = 30, *F*_(4,52)_, connection threshold: *p* < 0.05, FDR-corrected]. There was no significant effect. Nevertheless, as it can be seen in Figure 2B, all four ROI pairs followed a similar pattern in terms of their functional connectivity patterns. The anodal (solid line) and cathodal (dashed line) groups showed an opposing trend, where the anodal group exhibited heightened connectivity post-tDCS and lowered connectivity post-TMS, whereas the cathodal group showed decreased connectivity after the tDCS and increased connectivity after the TMS. A one-way ANOVA testing of the sham group measurements of the three timepoints did not show a significant change in activity throughout the experiment for any of the 10 connections.

To compare functional network connectivity between the treatment groups at each of the post-stimulation timepoints, multivariate *post-hoc* t-tests were calculated, and the total alpha error was kept at 5% using an FDR approach. Results are summarised in Table 3, Figures 2B, 2C. Post tDCS, functional connectivity differed between the anodal and the cathodal group, as well as between the anodal and the sham group. The anodal and the sham group did not show differential functional connectivity. The cathodal vs. anodal post tDCS contrast revealed two significant clusters comprised of lDLPFC to left and right temporo-parietal as well as striatal-right temporo-parietal and fronto-cingulate-left temporo-parietal cluster (connection cluster 1) and an lDLPFC to fronto-cingulate connection (connection cluster 2). The anodal vs. sham post tDCS contrast did show differences in terms of lDLPFC to fronto-cingulate connectivity. Post TMS, the anodal vs. cathodal contrast revealed two clusters comprised of a significant lDLPFC-to-fronto-cingulate link (connection cluster 1) and two lDLPFC to left and right temporo-parietal connections, as well as a striatal-left temporo-parietal link (connection cluster 2). The post-TMS cathodal vs. sham comparison showed lDLPFC connections to the fronto-cingulate and the striatum, respectively (connection cluster 1) and a fronto-striatal and a left and right temporo-parietal connection difference (connection cluster 2).

To summarise, in both post-stimulation rsfMRI sessions, functional network connectivity differed significantly between the anodal and the cathodal group. Post tDCS, the cathodal and the sham group did not differ, and post-TMS, the anodal and the sham group did not differ in terms of functional connectivity. In Table 3, significant connections that were not already revealed in the seed-to-voxel analysis are marked with an underscore. That is, post tDCS, the anodal-cathodal differences involved the striatal-right temporo-parietal and fronto cingulate-left temporo-parietal connection, whereas post-TMS, anodal-cathodal-differences involved the striatal-left temporo-parietal connection. Left-right-temporo-parietal and fronto-striatal connectivity only differed between the cathodal and sham group post-TMS. Figure 2C shows that those connections additionally found in the ROI-to-ROI-analysis did not show the hypothesised pattern as clearly compared to the ROIs linked directly to the stimulation site in our analysis (compare Figure 2B).

### Discomfort and blinding

Discomfort after TMS did not differ significantly between the tDCS groups (modal value = 4; Chi-square = 4.37, *p* = 0.11, df = 2; Kruskal-Wallis test). There was no significant difference in the SAM arousal ratings between the tDCS groups at any point of the experiment (Chi square_Baseline_ = 2.37, *p*_Baseline_ = 0.31, Chi square_preTMS_ = 0.11, *p*_preTMS_ = 0.95, Chi square_postTMS_ = 1.51, *p*_postTMS_ = 0.47, df = 2, Kruskal-Wallis tests). Nineteen participants (63.33%) were not able to accurately tell whether they received experimental or sham tDCS.

After the tDCS, subjects named and rated the strength (on a scale from 1 to 4) of sensations during tDCS stimulation. Only three participants reported pain sensation (average strength rating of *M* = 1). Sensation strengths ratings were low and the number of occurrences did not differ significantly for any type of sensation (chi-square goodness of fit test: Yates’ Ch-square = 1.7, Yates’ *p* = 0.95, *df* = 6). The most-reported sensations were burning (anodal: *n* = 5, *M* = 1.2; cathodal: *n* = 6, *M* = 1.83; sham: *n* = 9, *M* = 1), itching (anodal: *n* = 4, *M* = 2; cathodal: *n* = 8, *M* = 2; sham: *n* = 5, *M* = 1), pinching (anodal: *n* = 5, *M* = 1.6; cathodal: *n* = 4, *M* = 1.5; sham: *n* = 5, *M* = 1.2) and warmth (anodal: *n* = 1, *M* = 1; cathodal: *n* = 1, *M* = 1; sham: *n* = 4, *M* = 1.25).

## Discussion

In this study, we explored the possibility of preconditioning the prefrontal cortex. This was done by first administering excitatory, inhibitory, or sham tDCS and then facilitatory high-frequency rTMS (iTBS) to the lDLPFC. The tDCS was thus meant to prepare the network and possibly modulate the effects of TMS on the network. Even though testing the whole design did not yield significant results, a hypothesis-driven whole-brain analysis testing for an interaction effect of the tDCS experimental group (anodal vs. cathodal) and the TMS effect (pre vs. post-TMS) revealed three highly body-axial symmetric clusters of significant size. One cluster contained mainly voxels in the bilateral anterior cingulate and paracingulate gyri, as well as small portions of the bilateral caudate. The two other clusters covered mainly the insular cortices and operculum, as well as Heschl’s gyri, bilaterally. Because of their bilateral location, as well as their anatomical and functional characteristics, it seems plausible that functional connectivity to the lDLPFC was indeed modulated by the experimental stimulation procedure. All clusters that were directly linked to the stimulation site in our results showed the same activation pattern that was consistent with the notion of preconditioning. That is, the three groups started with similar functional connectivity to the stimulation site. Anodal tDCS then increased functional connectivity, while cathodal tDCS decreased functional connectivity of the stimulation site and the corresponding clusters. After TMS, this pattern got inversed, as the anodal group showed the lowest functional connectivity and the cathodal group ended up displaying the highest values. The sham group did not show a substantial change in functional connectivity throughout the experiment. These findings are consistent with the notion of homeostatic plasticity. That is, TMS applied to a system that was preconditioned shortly before by increasing or decreasing the membrane potential did have a very different effect on functional connectivity. Our results thus show, for the first time, that the principles of homeostatic plasticity and preconditioning, which have previously been demonstrated in the motor cortex (Lang et al., 2004; Siebner et al., 2004; Cosentino et al., 2012), can be transferred to the prefrontal domain, and possibly extended to the anterior cingulate and paracingulate cortex, as well as the insular, operculum and Heschl’s gyri.

The hypothesised and observed interaction effect between experimental groups and stimulation timepoints was strongest in the prefrontal-fronto-cingulate connection but also significant in the bilateral frontal-temporo-parietal connections. A similar pattern, even if only significant at the post-TMS measures, was found in prefrontal-striatal connectivity. These areas are commonly linked to motivation and emotion (cingulate cortex, striatum), somatosensory and auditory function (paracingulate, insular, operculum and Heschl’s gyrus) but also pain perception and evaluation (Sawamoto et al., 2000; Eickhoff et al., 2006; Wunderlich et al., 2011).

Emotions such as stress and anxiety have consistently been associated with an increase in frontostriatal connectivity (Arnsten, 2009), and psychological states are likely to have a strong influence on the effectiveness of neuro-stimulation and *vice versa* (Carnevali et al., 2020). The mere physiological perception of pain or other stimulation-related sensations can be ruled out as a cofounder to the post-TMS results since all three groups received the same treatment. We did not see a difference in arousal or pain ratings in our different tDCS groups, and most participants were not able to accurately guess which tDCS stimulation they received. The observed data is thus likely to be affected by the stimulation protocol and should be functionally meaningful.

Stimulating the frontostriatal network as part of the clinically very significant dopaminergic pathway is a common goal in depression and Parkinson’s treatment (e.g., Bouyer et al., 1984; Furman et al., 2011; Heller et al., 2013; Shine et al., 2013; Baggio et al., 2015; Carriere et al., 2015; Kang et al., 2016; Xu et al., 2016). Disordered fronto-cingulate connectivity has also been linked to attention and mood disorders like depression (Schlösser et al., 2008; Pizzagalli, 2011). Cho and Strafella (2009), for example, found an increase in dopamine release in the ipsilateral anterior cingulate and the orbitofrontal cortex following rTMS to the lDLPFC.

Subjecting the data to a *post-hoc* ROI-to-ROI analyses to further explore frontostriatal and communication between the clusters found in the interaction analysis did not yield a significant group effect for the whole design. However, we did find a non-significant pattern of frontostriatal functional connectivity in the whole design analysis, corresponding to the idea of a preconditioning effect. That is, functional connectivity between the lDLPFC and the striatum was highest in the anodal tDCS group directly after the tDCS, as compared to the results of other groups at that measurement. The planned *post-hoc* comparisons of frontostriatal connectivity were significantly different between the anodal and sham groups post-TMS. This pattern corresponds to results found by other groups that found a facilitating effect of cathodal preconditioning and an inhibiting effect of anodal preconditioning on motor evoked potentials (Lang et al., 2004; Siebner et al., 2004; Cosentino et al., 2012). There seems to be a corresponding mechanism when using functional connectivity as an outcome measure. However, it is unclear if functional connectivity as a pure measure of synchronised BOLD-activation is sufficient to characterise stimulation effects. Additional outcome variables such as neurotransmitter release in connected areas and short- and long-term functional parameters will be important to observe in methodological and clinical research.

An alternative interpretation of the pattern found is that the reversal of the effect from post tDCS to post TMS is not a result of the iTBS but rather due to a homeostatic effect of connectivity returning to its baseline state following a short-term tDCS evoked change. This would indicate that the tDCS and TMS did not interact at all. This is certainly a possibility that cannot be disproven with the present data, as we, unfortunately, did not include a tDCS-only group. Given that we do not see changes in fronto-cingulate or fronto-temporo areas in the tDCS, but only a decrease in frontal-precuneus connectivity in the tDCS sham group that received only iTBS, there seems to be no strong whole-brain effect of the sub-threshold iTBS. A decline in frontal-to-precuneus connectivity might be due to the declining attention of the participants over the course of the experiment, as the precuneus has been associated with attention alertness (Li et al., 2020). However, the fronto-cingulate cluster found in the interaction contrast showed a post iTBS level of functional connectivity that was well above the baseline level. An effect size of this magnitude would not typically be expected to result from 10-min tDCS at 1 or -1 mA only since its effect on functional connectivity has previously been shown to be quite subtle or limited to cortical areas. Adams et al. (2022), for example, applied tDCS at 1.5 mA for 20 min and found an increase in mean functional connectivity between the middle prefrontal cortex and the pallidum, the caudate, the insula and the putamen of only around 0.15. In this study, fronto-striatal functional connectivity was negatively correlated before stimulation and only increased to be slightly less negatively correlated (insula and putamen) or marginally above a zero-correlation (pallidum and caudate). We only found positive correlations before and after stimulation in the fronto-striatal system. However, the significance of functional connectivity’s strength in anti-correlations is not well understood and should be studied further.

The fact that we did not find significant voxels affected by only the iTBS in the sham condition is most likely due to the low number of subjects in each group (power limitations are discussed further below). However, this does not indicate that there is no iTBS effect. Because we used subthreshold iTBS, we did not expect strong effects that reach the statistical significance threshold required for a whole-brain analysis. Other studies investigating subthreshold iTBS effects usually only look at specific ROIs (Fierro et al., 2010; Tik et al., 2017; Alkhasli et al., 2019) or behavioural outcomes. Even though very few studies compare stimulation intensities, some show similar or more significant behavioural effects for subthreshold as opposed to suprathreshold iTBS, especially when used as a preconditioning stimulation (Padberg et al., 2002; Lee et al., 2021).

Interestingly some authors have linked lDLPFC stimulation to visual hallucinations (Blanke et al., 2000), while others showed a decrease in auditory verbal hallucinations after frontal-temporal tDCS treatment (Rashidi et al., 2021). In a study on psychosis that did not use brain stimulation, patients showed decreased connectivity between the bilateral Heschl’s gyri and the dorsal anterior cingulate cortex but increased connectivity between the planum temporale and the right dorsolateral prefrontal cortex (Yoon et al., 2015). In our study, both Heschl’s gyrus and planum temporale showed a similar pattern of functional connectivity to the DLPFC and the striatum and differed significantly between the anodal and the cathodal group both after tDCS as well as after TMS. The principle of modulating fronto-temporo-parietal connectivity with preconditioning, which was proven in this study, might be relevant for further research into hallucination and psychosis treatment.

Some authors argue that the mere peripheral stimulation administered by the different tDCS protocols can contribute to observed results, especially since the precise neuronal mechanisms of tDCS remain unclear (Asamoah et al., 2019). Some even state that the commonly used dose of 1 mV is too small to induce actual physiological changes in the brain (Vöröslakos et al., 2018). However, this was argued for the motor system, and the authors did not study functional network connectivity.

The main limitations of this study are the low number of participants per group and the resulting reduced statistical power due to the complex experimental design. Given the high level of variation in BOLD activity and the very high number of comparisons in the statistical analysis, increasing the number of observations would give clearer results. Additionally, we acknowledge that the choice of stimulation site, as well as stimulation protocols, is rather arbitrary due to the lack of previous studies on this particular area. Although we used a TMS protocol that has previously been shown to increase frontostriatal functional connectivity (Strafella et al., 2001; Alkhasli et al., 2019), it is not clear whether the tDCS and TMS protocols used in the present study are able to induce a strong short-term potentiation effect in the prefrontal cortex and its connected areas. Jamil et al. (2020), for example, showed that tDCS effects can differ greatly depending on whether they are observed directly post tDCS or 1 h later. We did include only healthy and young participants and were not aiming for long-term effects. However, the potential for the therapeutic use of preconditioning is very evident, as it was shown that cortical-subcortical communication can be modulated by non-invasive brain stimulation. It remains an important question whether preconditioning can help induce long-term changes in connectivity and activation levels that are needed in therapeutic settings. Different stimulation protocols, sites and timelines, as well as potential clinical outcome variables for different patient groups, such as patients with mood disorders or neurodegenerative disorders, should be studied systematically.

This study is the first to combine tDCS and rTMS to demonstrate, as a proof of principle, a preconditioning and modulatory effect of the lDLPFC and its connectivity to the anterior cingulate, paracingulate, insular cortex, operculum and Heschl’s gyrus Application of excitatory iTBS to a tDCS-inhibited prefrontal cortex yielded a stronger activity in terms of functional connectivity than excitatory iTBS to a tDCS-enhanced prefrontal cortex. Our results demonstrate a complex whole-brain impact of brain stimulation on functional connectivity, as well as the importance of the pre-existing state of neural networks on stimulation outcomes. This study might stimulate further research into preconditioning of the prefrontal cortex that may inform clinical treatment trials investigating protocols using DLPFC as a stimulation site for the treatment of depression. Yet, there needs to be more research into different stimulation protocols and the possibility of modulating the activity of connected sub-cortical areas, as well as potential therapeutic use.

## Data Availability Statement

The raw data supporting the conclusions of this article will be made available by the authors, without undue reservation.

## Ethics Statement

The studies involving human participants were reviewed and approved by the ethical committee of the RWTH Aachen University Hospital (EK 357/15). The patients/participants provided their written informed consent to participate in this study.

## Author Contributions

Theoretical considerations, experimental design and experimental plan were prepared by KS, FB, and FM. The experimental setup and data collection were conducted by KS and IA. Data management and processing, statistical analysis and manuscript preparation were carried out by IA. All authors contributed to the article and approved the submitted version.

## Funding

This work was supported by the START program of the Faculty of Medicine at RWTH Aachen University (grant number 12/16).

## Acknowledgements

We thank Meike Schulte for her valuable help during data collection and LOCALITE Biomedical Visualization Systems GmbH (Sankt Augustin, Germany) for technical support.

## Conflict of Interest

The authors declare that the research was conducted in the absence of any commercial or financial relationships that could be construed as a potential conflict of interest.

## Publisher’s Note

All claims expressed in this article are solely those of the authors and do not necessarily represent those of their affiliated organizations, or those of the publisher, the editors and the reviewers. Any product that may be evaluated in this article, or claim that may be made by its manufacturer, is not guaranteed or endorsed by the publisher.
